# The Challenging Treatment of Cisplatin-Resistant Tumors: State of the Art and Future Perspectives

**DOI:** 10.3390/molecules28083407

**Published:** 2023-04-12

**Authors:** Giulia Coffetti, Martina Moraschi, Giorgio Facchetti, Isabella Rimoldi

**Affiliations:** Department of Pharmaceutical Sciences, University of Milan, Via Venezian 21, 20133 Milano, Italy

**Keywords:** platinum (II) complexes, platinum (IV) complexes, anticancer agents, orphan tumors, resistance phenomena

## Abstract

One of the main problems in chemotherapy using platinum drugs as anticancer agents is the resistance phenomenon. Synthesizing and evaluating valid alternative compounds is challenging. This review focuses on the last two years of progress in the studies of platinum (II)- and platinum (IV)-based anticancer complexes. In particular, the research studies reported herein focus on the capability of some platinum-based anticancer agents to bypass resistance to chemotherapy, which is typical of well-known drugs such as cisplatin. Regarding platinum (II) complexes, this review deals with complexes in trans conformation; complexes containing bioactive ligands, as well as those that are differently charged, all experience a different reaction mechanism compared with cisplatin. Regarding platinum (IV) compounds, the focus was on complexes with biologically active ancillary ligands that exert a synergistic effect with platinum (II)-active complexes upon reduction, or those for which controllable activation can be realized thanks to intracellular stimuli.

## 1. Introduction

Cancer is one of the most lethal diseases, causing millions of deaths worldwide [1,2]. In particular, carcinogenesis, the process responsible for healthy cells’ transformation into tumoral cells, is characterized by multi-stage evolution: initiation, promotion, and the malignant transformation of cells and progression [3,4,5]. During this process, some DNA mutations occur, providing the cancer with different distinctive features, such as uncontrolled cell proliferation, replicative cell immortality, the circumvention of growth suppressors (p53, RAS), the induction of angiogenesis, resistance to cell death, activation to invasion, and metastasis [6,7,8]. The principal cancer treatments rely on surgical resection, radiotherapy, chemotherapy, immunotherapy, and targeted therapy, but usually combined therapy is the preferred choice. Indeed, after surgery, the patient often undergoes radio- or chemotherapy [9]. The most common chemotherapeutic agents are based on platinum complexes, among which the first-in-class drug is cisplatin [10,11,12]. Transport across the cancer cell membrane is the first step for cisplatin therapy to be successful. Cisplatin is a highly polar species, and its cellular accumulation generally occurs at a slower rate compared with that of other small-molecule anticancer drugs. Apart from passive diffusion, other non-saturable systems, such as fluid-phase endocytosis, may be involved in platinum drug uptake, and evidence for some active or facilitated transport has been identified over the years. Moreover, several experiments have demonstrated a direct connection, with platinum-containing compounds trafficking to copper transporters [13]. Subsequent studies have highlighted the role of Ctr-1 in the uptake of cisplatin analogs. Arnesano’s extensive work [14,15,16] showed that the role of Ctr-1 and the other proteins involved in copper metabolism is unambiguously connected to platinum drug movements across cell membranes. Upon aquation, cisplatin is activated, and it is responsible for DNA lesions due to the formation of intrastrand crosslinks; this activates signal transduction pathways, leading to apoptosis [13,17]. On the other hand, it is also responsible for numerous side effects and, above all, drug resistance, inducing therapeutic failure despite consistent initial response rates [18,19]. Three mechanisms define cisplatin’s direct involvement, demonstrating the chemo-resistant tumor cell phenotype: pre-target, on-target, and post-target resistance [20,21,22]. The first is characterized by reduced intracellular accumulation and increased cisplatin sequestration caused by GSH and other scavengers. For example, recent metabolomics studies have revealed that the presence of the enzyme glucose-6-phosphate dehydrogenase (G6PDH) in cisplatin-resistant ovarian cell lines is responsible for NADPH production, which maintains the GSH level and redox balance, thus reducing cisplatin’s effectiveness [23]. In the second, activating particular classes of DNA polymerases removes platinum–DNA adducts that would normally generate an apoptotic signal. Moreover, multidrug resistance-associated proteins (MRPs) and ATP-binding cassette (ABC) transporters can also induce cellular resistance to cisplatin. MRP2 is mostly responsible for this effect, as its levels are overexpressed in patients with colorectal, hepatocellular, and esophageal cancer. Finally, after the interaction between cisplatin and DNA takes place, the third resistance mechanism involves defects in the signal transduction pathways that usually bring about cell death [24,25]. This is the main subject driving research toward developing new derivative complexes that can overcome these resistance mechanisms. For example, an emerging approach relies on introducing different metals. A vast library of non-platinum metal complexes has been prepared and evaluated for anticancer activities for this purpose. Ruthenium is one of the most investigated non-Pt metals for cancer treatment: Ru(III) complexes, especially NAMI-type ones, are useful for their excellent antimetastatic properties [26]. Moreover, polypyridyl Ru(II) complexes have been extensively investigated as a possible DNA “light switch”, with interesting photophysical and cell migration inhibition properties [27]. Another promising strategy to improve the anticancer efficacy of platinum drugs and overcome their shortcomings is incorporating a second metal center with distinct biological targets, oxidation states, and ligand(s) of different natures into platinum complexes, which could influence their modes of action [28].

Additional studies have led to the discovery of a second and third generation of platinum complexes. Respectively, these are carboplatin, which possesses higher solubility and stability than cisplatin, and oxaliplatin, which is water-soluble and exhibits a broader spectrum of activity than other complexes.

Starting from cisplatin’s structure, some ligand modifications that could improve the complex’s biological properties have been introduced [29,30].

**Figure 1:**
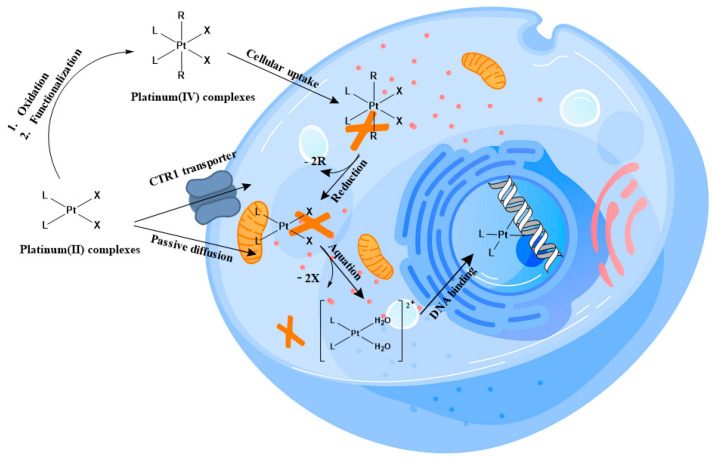
Pt(II) and Pt(IV) trafficking within cells.

This review aims to go through all the progress of the last two years regarding the design of platinum complexes, both as Pt(II) and Pt(IV), used as anticancer agents in the treatment of cisplatin-resistant and orphan tumors (Figure 1).

## 2. Treatment of Cisplatin-Resistant Tumors: Pt(II) Complexes

### 2.1. Pt(II) Complexes in Trans Conformation

**Figure 2:**
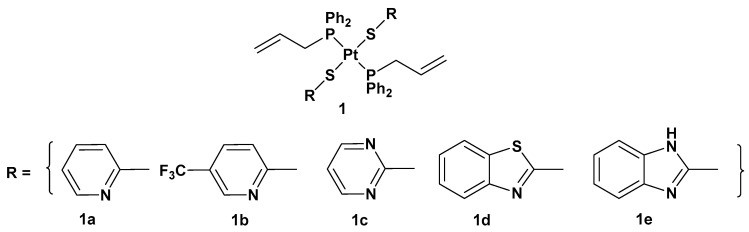
*Trans*-Pt(II) complexes with heterocyclic thionate ligands **1a**–**1e**.

There are different ways to overcome cisplatin drug resistance, side effects, toxicity, and poor selectivity. One of them is related to the introduction of a trans conformation of Pt(II) complexes. The advantage of this approach is that there are different structural and DNA-binding properties in comparison with cisplatin and its derivatives, which notably possesses a cis arrangement of ligands around the metal center. For this reason, a series of trans-Pt(II) complexes (complex **1**) with heterocyclic thionate ligands has been synthesized (Figure 2) [31].

**Figure 3:**
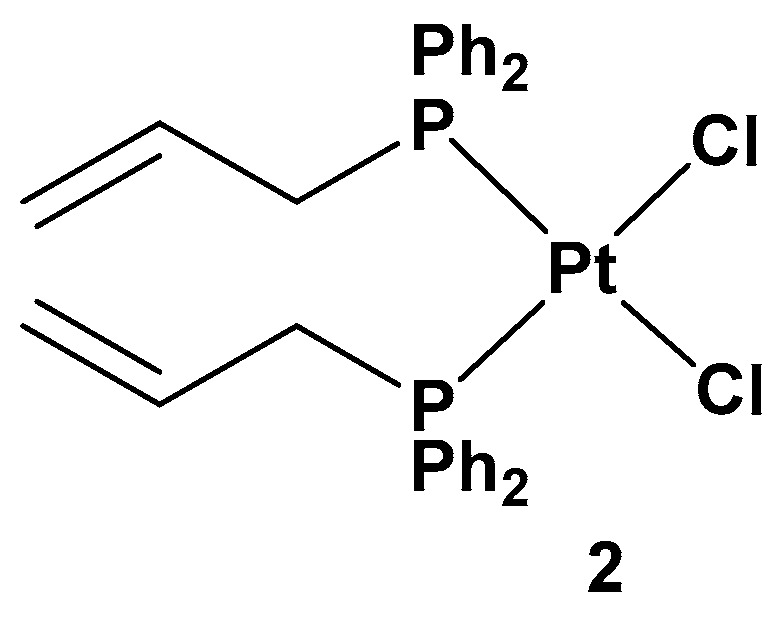
Dichloride *cis*-Pt(II) complex (complex **2**).

All of these new complexes have been tested on three human cancer cell lines (A549 lung, SKOV3 ovarian, and MCF-7 breast cancer cell lines) and studied for their antiproliferative activities in comparison with cisplatin and complex **2**, a dichloride Pt(II) complex counterpart in a cis conformation (Figure 3).

All the new complexes showed cytotoxicity in vitro against the three cancer cell lines, but only complex **1a** showed statistically significant different activity compared with cisplatin, with significantly lower IC_50_ values, 4.31, 6.23, and 4.80 μM, compared with 9.71, 14.48, and 11.59 μM for cisplatin against A549, SKOV3, and MCF-7, respectively. Moreover, and most importantly, all complexes showed selectivity between tumorigenic and nontumorigenic cell lines. Interestingly, complex **2** showed lower antiproliferative activity (22.49, 34.50, and 19.01 μM against A549, SKOV3, and MCF-7, respectively) than others; complex **1b** and, above all, **1a** showed an ability to induce apoptosis in cancer cells with the highest potency, such that **1a** has been used as the representative compound of the series in further studies. In addition, **1a** can target the genomic content of MCF-7 cancerous cells and directly interact with DNA, as confirmed by comet assay. However, further studies are needed to identify the underlying mechanism of action of these platinum drugs within biological systems.

**Figure 4:**
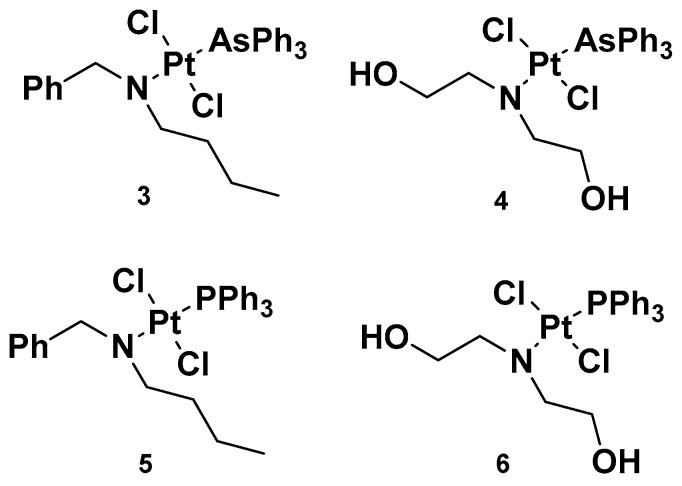
Complexes **3** and **4** and previously synthesized analogs **5** and **6**.

Analogously to complexes **1a**–**e**, trans-Pt(II) complexes with triphenylphosphine ligands have also been synthesized, and in particular, complexes **3** and **4** have been evaluated for their promising anticancer activities (Figure 4) [32]. Indeed, several analogs have been studied, starting with complexes **3** and **4,** where a triphenylphosphine ligand was substituted with a triphenylarsine ligand (complexes **5** and **6**) with the same dialkylamino ligand preserved, in addition to the two chloride ligands. For all the performed studies, cisplatin was used as a reference and **3** and **4** were used as comparisons.

Complex **3**′s results were the most effective due to the presence of N,N-dialkylamino side chains, which contributed to an increase in lipophilicity in the complex. This is an important aspect to evaluate, as it could be useful in overcoming one of the primary issues involved in cisplatin resistance, i.e., low cellular accumulation. The Pt content found in cells incubated with **3** was found to be higher than the reference drug, suggesting the ease **3** has in crossing the cell membrane.

Furthermore, in comparison with complex **5**, complex **3** has a higher cytotoxic effect: the IC_50_ values were 0.49 and 1.85 μM compared with 3.55 and 3.63 μM, seven and two times lower than complex **5** in sensitive and resistant ovarian carcinoma cells, A2780 and A2780cis, respectively. These results underline that the triphenylarsine group is more effective than the ancient triphenylphosphine group in eliciting cytotoxicity.

In studying platinum complex interactions with salmon testis DNA, complex **3** showed the ability to covalently link with the target, but to a lesser extent in comparison with the reference drug, cisplatin. In addition, this cytotoxicity was related to the ability of complex **5** and complex **3** to interfere with the activity of topoisomerase II, an important enzyme involved in DNA metabolism and correct functionality. In particular, **3** behaved as a topoisomerase II poison. This detrimental effect on topoisomerase II, along with its ability to induce transmembrane depolarization in mitochondria, is primarily responsible for the cytotoxic effect of the complex, and it can promote cell death through the apoptotic pathway.

**Figure 5:**
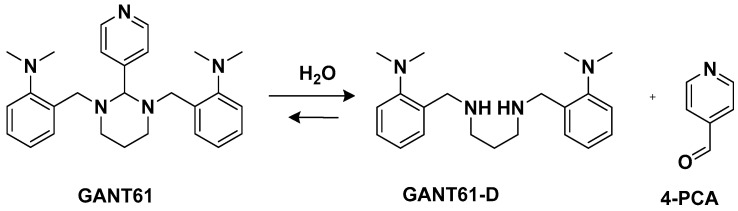
GANT61 hydrolysis releasing GANT61-D and 4-PCA.

Another trans-conformation Pt(II) complex that can be used to overcome cisplatin resistance and its side effects is an inhibitor of the Hedgehog (Hh) pathway based on GANT61 (Figure 5) [33]. The Hedgehog signaling pathway regulates cell differentiation, cell proliferation, and stem cell maintenance during embryonic development, which results in the transcription of three glioma-associated oncogene homolog (GLI) transcription factor proteins: GLI1, GLI2, and GLI3. Thus, inhibiting this pathway means targeting cancer stem cells (CSCs). GANT61 could be useful for this because it can inhibit the Hh pathway at the GLI level, and it shows antiproliferative activity in vitro and in vivo. In vivo, it undergoes hydrolysis to produce 4-pyridine carboxaldehyde (4-PCA) and the bioactive diamine derivative GANT61-D.

**Figure 6:**
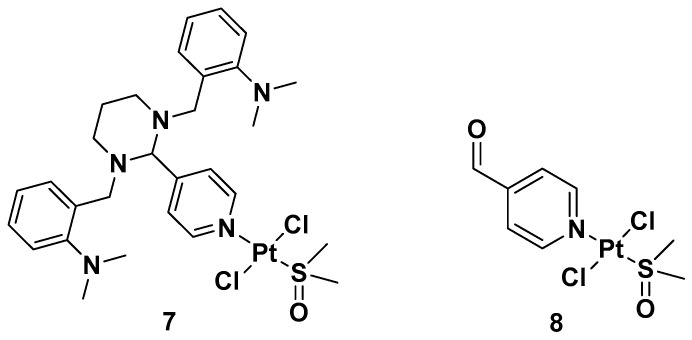
Complexes **7** and **8**.

Two *trans*-[Pt(II)Cl_2_(dmso)L]-type complexes, **7** and **8**, where L represents both GANT61 and 4-PCA, have also been synthesized (Figure 6).

Both complexes have been tested on two human mammary epithelial cell lines, HMLER and HMLER-shEcad. This showed a larger cancer stem-like cell (CSC) population, using salinomycin as the positive control and cisplatin and carboplatin as the Pt compound control. Complex **7** showed the highest potency against both cell lines (the IC_50_ values were 1.0 and 2.6 μM against HMLER and HMLER-shEcad cell lines, respectively) but without selectivity for the CSC-enriched cells and normal cells. It also showed positive activity against mammospheres, the multicellular, three-dimensional structures formed by CSCs: a significant reduction in their formation, size, and viability could be observed. Complex **7** induced apoptosis against two triple-negative breast cancer (TNBC) cell lines, MDA-MB-231, and BT549, with a potency that is, respectively, thirty and three times higher than complex **8** (IC_50_ values of 3.6 and 4.0 μM for complex **7** vs. IC_50_ values of >100 and 12.78 μM for complex **8** against MDA231 and BT549, respectively). A possible explanation for this activity is related to the presence of GANT61 as an N-donor ligand in complex **7**; according to the abovementioned hydrolysis process (Figure 4), it releases the bioactive Hh pathway inhibitor GANT61-D, and 4-PCA can remain bound to the Pt(II) center. In addition to inhibiting the Hh pathway, complex **7** can bind DNA, as shown by an increase in the DNA damage marker γH2AX and a reduction in the expression of GLI-1 and GLI-2, confirming the GLI function in the nucleus is blocked.

### 2.2. Pt(II) Complexes Carrying Biologically Active Ligands

Since the aim is to improve the anticancer effects of conventional platinum-based drugs, another possible strategy is selectively introducing structural changes around the active platinum center by choosing either carrier ligands endowed with biological activity by themselves or substituting common chloride ligands.

**Figure 7:**
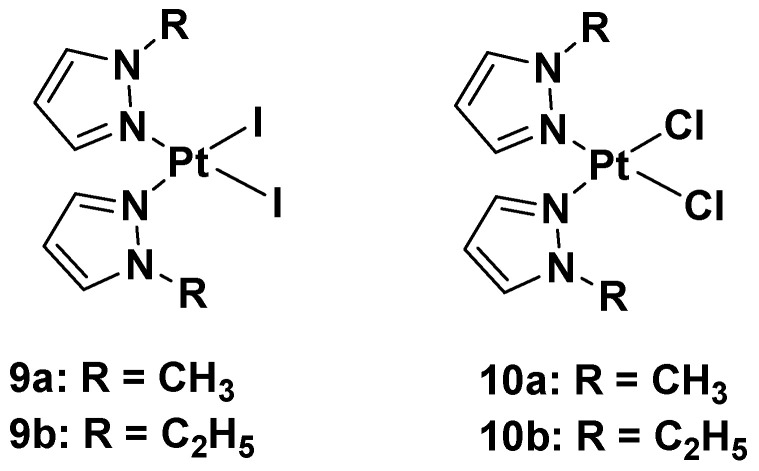
Platinum(II) diiodide complexes and platinum(II) dichloride complexes.

In this case, new platinum(II) complexes with 1-alkyl-1H-pyrazole ligands containing both iodide- and chloride-leaving groups have been synthesized (Figure 7) [34].

It was discovered that both iodide complexes, **9a** and **9b**, were more effective than the chloride analogs, **10a** and **10b**, and cisplatin against all cisplatin-resistant cell lines; while the chloride complexes had an effect comparable to cisplatin: 11 and 11.9 μM vs. 4 μM in the A2780 cell line; 21 and 19 μM vs. 22 μM in the A2780cisR cell line; 29 and 25 μM vs. 24 μM in the MDA-MB-231 cell line. These differences show that the mechanism of action of the new alkyl pyrazole complexes is different from that of cisplatin, thus potentially allowing the compounds to overcome the resistance mechanism. Another important aspect is that the new Pt(II) complexes are more effective in tumor cells with respect to healthy cells, thus confirming their higher selectivity toward tumor cells. Coherent with the antiproliferative effect, the intracellular Pt amount increases with lipophilicity: the most lipophilic complex causes the ethyl group to bond to the pyrazole and the iodide-leaving ligands. However, even if the amount of Pt from complexes **9a** and **b** was about ninety-fold higher than cisplatin, their effectiveness would only be five-fold higher than that of cisplatin (4.2 and 5.0 μM vs. 24 μM in the MDA-MB-231 cell line). This means that a higher amount of Pt(II) alkyl pyrazole complex is necessary to obtain a certain level of biological activity compared with cisplatin.

These data suggest a new anticancer mechanism compared with cisplatin, whose main target is nuclear DNA. Binding studies using calf thymus DNA have revealed that both chloride and iodide complexes can covalently interact with the target, but although iodide binds DNA more efficiently than its chloride analog (as demonstrated by ICP-MS), the DNA platination occurs in both to a lesser extent than cisplatin. These results suggest that DNA might not be the main target of the new complexes, **9a**,**b** and **10a**,**b**. The experimental evidence shows that the contribution exerted by the unrepaired DNA lesions (due to alkyl pyrazoles) on cytotoxicity was minimal compared with cisplatin. Conversely, it was found that the time-dependent cell response profiles (TCRPs) for complexes **9a**,**b** and **10a**,**b**, which can predict the mechanism of action in biologically active compounds, significantly differed from those typically expressed by DNA-damaging agents, such as cisplatin. Their profiles were instead superimposable on those displayed by nontubulin-targeting mitotic inhibitors toward Eg5. The mitotic tubulin Eg5 is an essential spindle motor protein, and it assembles and maintains the bipolar spindle during mitosis. When inhibited, a monoastral spindle forms, thus resulting in mitotic arrest. In the literature, it is reported that pyrazoles are important and potent inhibitors of Eg5, so analyzes have been performed to ascertain whether all the new Pt(II) complexes, **9a**,**b** and **10a**,**b**, cause a cell cycle arrest in the G2/M phase. This plausible molecular target also explains the selectivity for tumor cells exerted by these complexes, since healthy cells do not divide or only slowly proliferate tumor cells.

**Figure 8:**
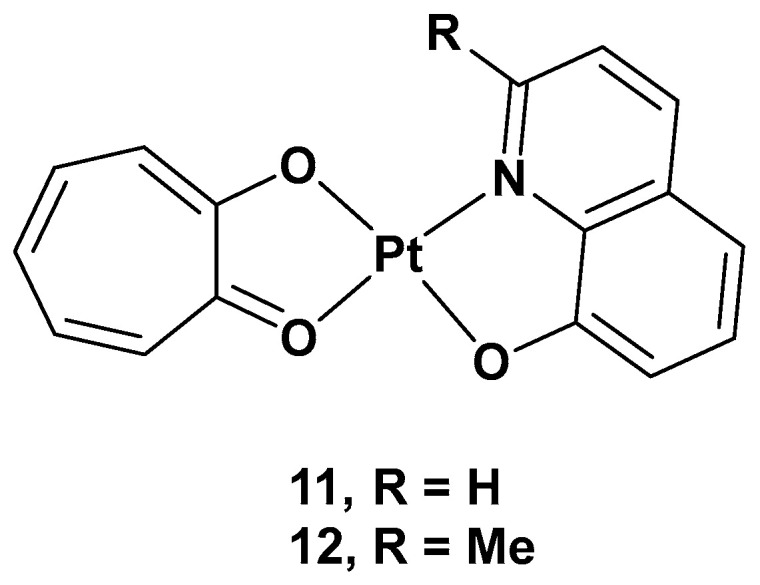
[Pt(Q)(L)] (**11**) and [Pt(MQ)(L)] (**12**).

Concurrently, to develop platinum complexes able to hit novel molecular targets, one possible strategy relies on synthesizing Pt(II) complexes based on natural products, which have been recognized for their biological activity. Tropolone derivatives could be a significant example consistent with this idea [35]. Tropolone is a natural product isolated mostly from plants and fungi, with a seven-membered aromatic ring possessing strong activity against bacteria, antibiotic-resistant bacteria, and fungi. The different substitution groups on the aromatic ring could confer it with a plethora of different properties, such as antiviral, anti-inflammatory, and antidiabetic activities. Thus, two new complexes, [Pt(Q)(L)] (**11**) and [Pt(MQ)(L)] (**12**), were synthesized, and their cytotoxicity was evaluated, in which tropolone was combined with 8-hydroxy-quinoline (Q) or 2-methyl-8-hydroxy-quinoline (MQ), two pyridinic scaffolds known for their cytotoxic activity in different types of tumor cell lines (Figure 8).

The cytotoxicity was tested on different cancer cell lines: HeLa, T24, A549, NCI-H460, and MGC80-3, as well as on HL-7702 (a healthy cell line). Complex **11** showed high cytotoxicity in these cell lines, especially T24 (IC_50_ = 3.6 μM), but also significant cytotoxic activity against the normal cell line, HL-7702; complex **12** showed selectivity in the tested cell lines, such as T24 and MGC80-3 (the IC_50_ values were 10.3 and 5.97 μM, respectively), and lower cytotoxicity than cisplatin in the normal cell line. Overall, complex **11** showed an effect against T24, while complex **12** showed effects against both T24 and MGC80-3. The possible anticancer mechanism of both complexes, but especially complex **11**, may be the production of high ROS levels through cell mitochondria, which can damage organelles and induce severe dysfunction in the cells’ machinery. In addition, complex **11** was proven to induce apoptosis by decreasing mitochondrial membrane potential, with a simultaneous increase in the Ca^2+^ level in the cells contributing to the onset of mitochondrial dysfunction, and, finally, by activating caspase-3 and caspase-9. Furthermore, complex **11** induced cell death in the G2 phase, downregulating the expression of Cdc25A, CDK1, cyclin A, and cyclin B and upregulating the expression of p27 and p21, proteins that interact with cyclin–CDK complexes within the nucleus and, hence, modify cell cycle progression. On the contrary, complex **12** induced cell arrest in the G1 phase and had similar effects to **11**, but it lacked the influence of p27 expression. Complex **11** showed a significant in vivo anticancer effect, revealed by its inhibiting effects on tumor growth in T24 xenograft mice without any renal pathological changes or influence on renal function.

**Figure 9:**
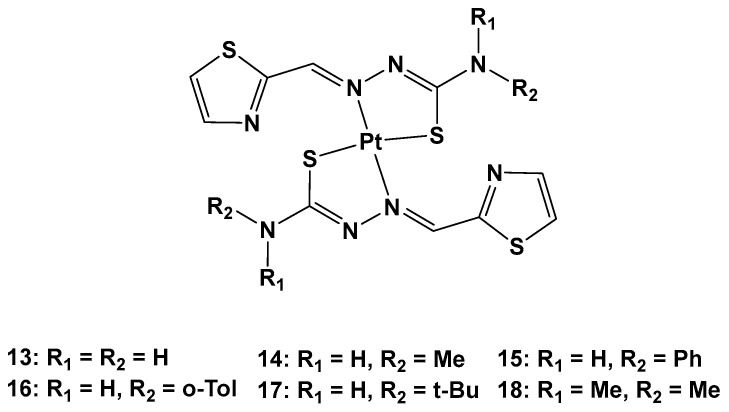
Series of new Pt complexes, **13**–**18**.

Furthermore, thiosemicarbazones have shown promising activity for cancer therapy by inhibiting P-gp expression in particular. Considering the beneficial effects on both the pharmacokinetics and pharmacodynamics arising from metal coordination, their platinum complexes also possess promising biological activities, such as anticancer, antibacterial, and antiviral properties. For this reason, six novel Pt(II) complexes (**13**–**18**) were synthesized to overcome cisplatin resistance (Figure 9) [36].

The antiproliferative activity of **13**–**18** was then evaluated using an MTT assay. It was discovered that the anticancer effect was improved when R_1_ was a methyl (**14**), phenyl (**15**), *o*-toluene (**16**), or tert-butyl (**17**), and the best activity occurred when both R_1_ and R_2_ were methyl groups. Furthermore, the different substitutions of R_1_ and R_2_ also influenced the accumulation of Pt agents in cancer cells, such that the anticancer activity could be modified by regulating the lipophilicity of the ligands.

To confirm the ability of these complexes to overcome cisplatin resistance, they were analyzed in comparison with cisplatin. After 48 h of incubation, the antiproliferative activity of these Pt complexes is two- to seven-fold higher than the activity of cisplatin against cisplatin-resistant lung cancer (A549cisR) cells (IC_50_ values from 5.02 to 15.32 μM vs. cisplatin value of 36.58 μM). The higher concentration of Pt inside the cells correlates with higher anticancer activity.

Both **13** and **18** can induce cell apoptosis in the depths of the 3D cell spheres, and **18** has a greater effect on metastasis than cisplatin or **13**. This means that **18** shows not only an improved tumor penetration potential, but also effectiveness in inducing cell death. Complex **18** can bind to DNA, leading to the formation of DNA-Pt adducts that can form inter-strand crosslinks, arresting DNA synthesis. It can also induce mitochondrial apoptosis and lethal autophagy.

However, the principal mechanisms by which **13** and, above all, **18** overcome cisplatin resistance rely on three different possible mechanisms: the inhibition of endogenous P-gp expression, a transporter that enables the active efflux of the drug from the cancer cells; the generation of ROS inside the cells that can reduce GSH levels, a reducing substance; and the inhibition of the MEK/ERK pathway. Another advantage of **18** is its ability to avoid important side effects in vivo, i.e., there has been no significant body weight loss observed in treated mice.

**Figure 10:**
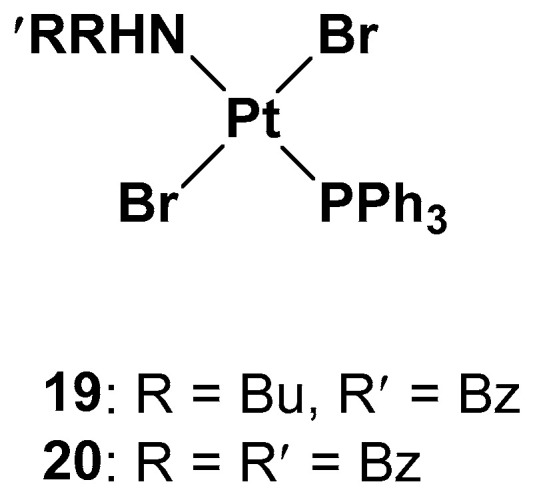
Pt(II) complexes **19** and **20**.

Since one of the chemoresistance mechanisms is represented by the overexpression of enzymes in the thiol redox system, such as glutathione and thioredoxin, which are generally involved in the regulation of redox balance in cells, two new Pt(II) complexes containing the triphenylphosphine moiety (widely used as a mitochondriothropic moiety), the dialkylamino group, and the bromide groups were synthesized (Figure 10) [37].

It is worth noting that the substituents of the amino group are fundamental to the cytotoxicity of both **19** and **20** in resistant cell lines, but, at the same time, complex **19** has proven to be more effective, underlying the beneficial effects of the dissymmetrical substitution pattern between these two brominated triphenylphosphine trans-platinum derivatives (the IC_50_ values are three times lower than the cisplatin values, 2.15 μM vs. 6.61 μM, respectively).

While studying the mechanism of action of these two complexes, some differences with cisplatin have been found. First of all, from the cell uptake point of view, after 60 min of incubation, the amount of Pt is comparable with cisplatin and **19**; after 180 min, the uptake of cisplatin does not change, but the amount of complex **19** increases threefold. Indeed, while cisplatin probably undergoes an efflux effect, compound **19** shows a time-dependent accumulation in cells and a greater ability to cross the membrane. DNA interaction has been confirmed for both complexes due to the presence of the triphenylphosphine hydrophobic moiety and the trans geometry of bromide groups. However, this is not the only target that could explain the cytotoxic effect. Different biological assays support complex **19**′s ability to depolarize more mitochondrial membranes than cisplatin under the same concentrations and experimental conditions, thus confirming that it interferes with the cellular redox state. Both complexes **19** and **20** can decrease the total thiols in both cisplatin-resistant and -sensitive cell lines, with cisplatin influencing the thiol concentration to a lower extent and only in the sensitive cell line. In addition, another mechanism of cytotoxicity is related to its influence on thioredoxin reductase (TrxR) activity. TrxR, along with glutathione reductase (GR), is a key enzyme in redox regulation and antioxidant responses inside cells. High levels of TrxR are one of the main characteristics of cisplatin-resistant tumor cells that need to be defeated. In cisplatin-resistant cells, complexes **19** and **20** can both affect the responsivity of TrxR, resulting in a lower amount than in untreated cells.

### 2.3. Ionic Pt(II) Complexes

**Figure 11:**
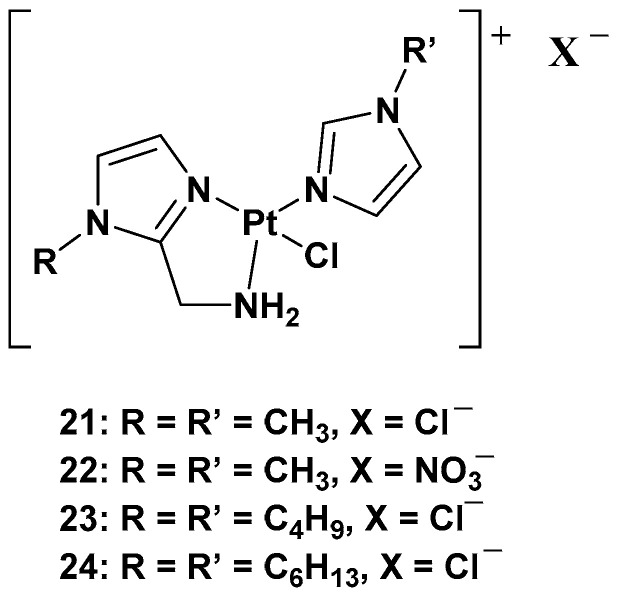
Series of cationic platinum(II) complexes, **21**–**24**.

The purpose of synthesizing monofunctional cationic Pt(II) complexes is to produce substrates that benefit from binding to organic cation transporters (OTCs). This allows them to be used in the selective treatment of tumors where this transporter is overexpressed. Indeed, most of these transporters display high tissue specificity and subcellular or peculiar expressions, thus translating into specific tumor efficacy. To reach this goal, a series of cationic triamine platinum compounds have been synthesized. The general formula is [Pt(N-N′)N′Cl]X, where N–N′ is an aminomethyl–imidazole ligand, and N′ is an imidazole ring bearing the same alkyl group in the N1 position (Figure 11) [38,39,40].

**Figure 12:**
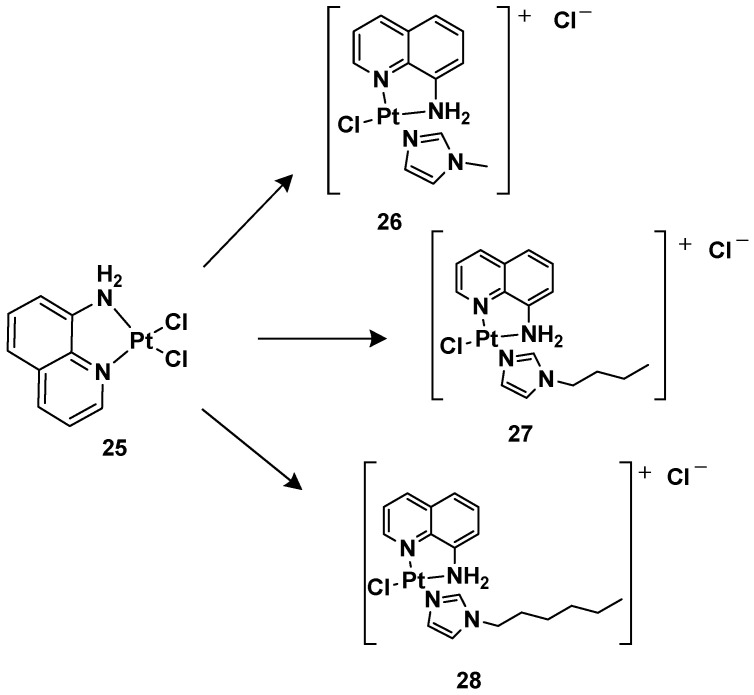
Synthesis of cationic platinum(II) complexes, **25**–**28**.

After this series, where complex **24** is more effective compared with cisplatin, other ligands have also been studied, such as 8-aminoquinoline and its chiral 5,6,7,8-tetrahydro-derivatives, also called CAMPY (Figure 12) [41].

Modifying the ligand causes a different biological effect in the corresponding cationic platinum(II) complexes. These differences are principally found in the accumulation of the complexes in different cellular phases, which results in a different effect on DNA: they seem to arrest cells into a G0/G1 phase to a greater extent; they more efficiently induce p53 mRNA; and α-tubulin and β-actin levels significantly reduce in response to new platinum(II) complexes. Furthermore, it should be highlighted that **28** is the most potent cytotoxic agent of this series against the triple-negative breast cancer MDA-MB-231, a particularly aggressive tumor still lacking effective chemotherapeutic treatments.

For this reason, **28** (**Pt-8AQ**) has been further analyzed against six tumor cell lines. Three cell lines of human glioblastoma were present, i.e., U87-MG (expressing wild-type p53), U373-MG, and U138-MG (expressing endogenous mutant p53) [42].

Complex **28** shows higher activity against glioblastoma cells compared with cisplatin, with IC_50_ values of 3.68, 11.53, and 8.05 μM against U87-MG, U373-MG, and U138-MG, respectively, compared with cisplatin, which shows IC50 values of 7.27, 22.69, and 32.1 μM against U87-MG, U373-MG, and U138-MG, respectively, while they had a similar effect on pancreatic adenocarcinoma CFPAC-1, mesothelioma MSTO-211H, and adenocarcinoma MCF-7. Furthermore, a comparison relative to the stability of the complex compared with cisplatin was made after incubating them at 37 °C for 24 h. Interestingly, **28** showed only 2.05-fold decreased activity, but significant pharmacological activity was still present. Furthermore, it was impossible to measure the IC_50_ of the cisplatin, thus indicating that the activity was completely lost after the treatment. In addition, the authors proved that even after 6 days of treatment, **28** maintained some pharmacological activity, paving the way for the development of slow-release chemotherapeutics.

To understand this new complex’s mechanism of action, its interaction with some possible molecular targets and nucleophiles known to prevent platinum-based chemotherapy was studied. One of the most important factors responsible for drug resistance is glutathione (GSH) and, through ^1^H-NMR analysis, it was possible to confirm the interaction with **28**; other factors are the membrane transporters and channels, called transportomes, in which copper transporters (CTRs) important for the cellular uptake of platinum are present. Furthermore, in this case, a monocoordinated complex with a 1:1 stoichiometry was found using ESI-MS experiments by exploiting the peptide Mets7, which mimics the methionine-rich motif involved in platinum coordination. Similarly, the interaction with 9-ethylguanine (9-EtG) was confirmed via ^1^H-NMR, and this result highlights that DNA is the main target of this complex. Furthermore, the cytotoxicity of **28** up to a concentration of 20 µM against all three human glioblastoma cell lines is higher than that of cisplatin, whose IC_50_ was undetectable. The new complex affects the cells in the G0/G1 phase, showing an earlier cell death mechanism. Since the three cell lines differ from each other regarding the genetic status of p53, the influence of **28** and cisplatin on the functional expression of this gene was evaluated, showing a different impact on the proapoptotic molecules BCL-2, BAX, PUMA, and NOXA. Both complexes downregulate p53, except in U138-MG upon treatment with **28**, and BCL-2 is downregulated by both complexes in all cells. In U87-MG, cisplatin upregulates PUMA, BAX, and NOXA. However, **28** upregulates NOXA in a more significant way, though BAX is downregulated, whereas PUMA levels remain unaltered. In U138-MG, the three genes are downregulated by cisplatin and upregulated by **28**; in U373-MG, all three genes are downregulated by both complexes.

To confirm these cytotoxic effects, photographs under a light microscope were taken and a triple staining assay was performed in one study. In cells treated with cisplatin, there were no consistent changes compared with the untreated cells, while cells treated with **28** detached from the surface and completely changed in morphology, which is typical of damaged cells. In contrast, the control cells were viable and adherent, cells treated with cisplatin were early-apoptotic, and cells treated with **28** were mostly detached and late-apoptotic. Interestingly, there were no necrotic cells, thus indicating that apoptosis is the exclusive cell death mechanism.

These experiments confirmed that **28** is a promising candidate for treating human glioblastoma, and considering its high chemical stability, it may be suitable for loading on mesenchymal stromal cells (MSCs) as a new drug delivery system [43,44,45,46,47,48]. MSCs are cells from different tissues that can uptake drugs and release them directly into the neoplastic microenvironment due to their ability to accumulate in the stroma of several primary and metastatic neoplasms. This system aims to target the tumor cells, reduce the side effects, and overcome cisplatin resistance [49].

**Figure 13:**
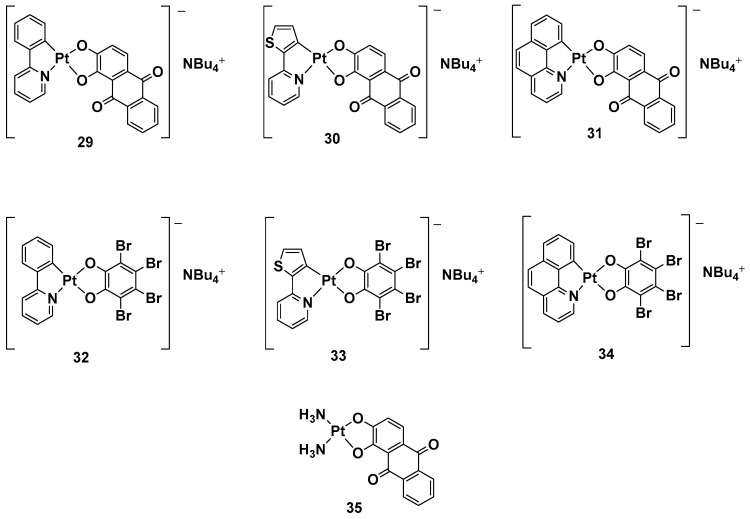
Anionic Pt(II) complexes, **29**–**35**.

While a plethora of cationic platinum complexes has been proposed in recent research, anionic Pt(II) complexes have never been tested for their anticancer activity. A series of complexes with the general formula NBu_4_[(C^N)Pt(O^O)] has recently been synthesized and evaluated for promising TNBC activity and theranostic properties (Figure 13). The structure of this series can comprise a C^N ligand, which is a cyclometalated form of 2-phenylpyridine (H(PhPy)); 2-thienylpyridine (H(Thpy)); or 2-benzo[h]quinoline (H(Bzq)), whereas the dioxygenated leaving group O^O represents the dianion of tetrabromocatechol (H_2_(BrCat)) or the dianionic form of alizarine (H_2_(Aliz)) [50].

Alizarine moiety, because of its extended aromatic system, was chosen to introduce a labeling system to the anticancer activity, furnishing the properties of the emissive compounds so that, in principle, many biologically relevant processes could be followed in real-time by Pt-based probes. All these new complexes were tested against TNBC, an “orphan” tumor that has a negative response to currently approved drugs and hormonal therapy, as well as compounds that target HER2 protein receptors; in particular, its in vitro biological activity against the MDA-MB-231 cell line was tested.

Complexes with bromocatechol (**32**–**34**) showed good cytotoxic activity against MDA-MB-231 cells, which were independent of the cyclometalated ligand’s nature. Complexes **29**–**31**, possessing alizarine as a ligand, were studied as a mixture of the two isomers, showing a different form of cytotoxic activity that depends on a different C^N ligand. In particular, complex **29** possessed PhPy, which showed the highest cytotoxic activity (IC_50_ = 1.9 μM) and selectivity between the tumoral cells and nontransformed vascular smooth cells (IC_50_ = 12.5 μM vs. 1.9 μM). As to whether this activity is independent of the deoxygenated ligand, complex **35** was chosen as a test bed to confirm this hypothesis. Substituting the cyclometalated ligand with two ammonias dramatically reduced the cytotoxic activity in comparison with both its cyclometalated counterparts and cisplatin, confirming phenylpyridine and alizarine are the best matches for exerting a maximum anticancer effect. Furthermore, studying the effect of the new complex on the cell cycle progression showed that complex **29** displays antiproliferative activity, blocking the progression of the cells through the S phase and decreasing cells in the G2/M phase.

In addition, even if all the anionic complexes were found to be emissive in solution in the red region of the electromagnetic spectrum in a confocal analysis, it was possible to localize complex **29** in the perinuclear region.

## 3. Treatment of Cisplatin-Resistant Tumor: Pt (IV) Complexes

Platinum(IV) complexes are prodrugs that have to be activated into platinum(II) complexes to act as anticancer agents. The anticancer potential of platinum(IV) agents has been known for many years, but their potential anticancer activity still needs to be elucidated and investigated; even if some complexes show encouraging pharmacological profiles, reaching different clinical phases of evaluation, they have not been approved for clinical use yet [51].

These new complexes are six-coordinated and possess octahedral geometry that can confer better stability than the square-planar geometries of platinum(II) complexes. Indeed, the lack of empty space within the platinum coordination sphere ideally prevents the metal in its +4 oxidation state from being attacked by nucleophilic substitution in the circulatory system, endowing Pt(IV) derivatives with an innate advantageous inertness over the Pt(II) counterparts.

**Figure 14:**
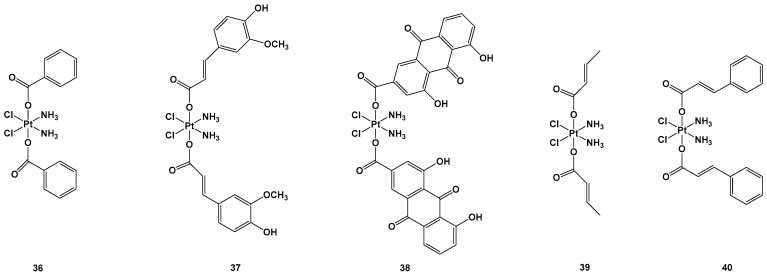
Pt(IV) complexes **36**–**40**.

The synthesis of platinum(IV) complexes instead of platinum(II) complexes could lead to further important advantages, such as lipophilicity, stability, the possibility of oral administration, cell targeting, and improved cellular uptake [52]. Another advantage is the possibility of enhancing the therapeutic effect, conjugating active biological moieties in the axial position to the center of the platinum. The ligands in the axial position have to be released to activate platinum(IV) into the reduced active form, which carries a platinum(II) center. This has a favorable synergistic effect on the tumoral cells. Octahedral platinum(IV) complexes are nontoxic to cancer cells on their own; they require a bioreduction process in their activated forms, and this is why they are considered prodrugs [53]. Some recent examples clarifying this strategy are reported in [54]. Two Pt(IV) complexes, in which the ligands were ferulic acid (FA-COOH) and rhein (RH-COOH) and complexes **37** and **38,** were synthesized and compared with other complexes in which the ligands were benzoic acid (BA-COOH), crotonic acid (CA-COOH), and trans-cinnamic acid (TCA-COOH) and complexes **36**, **39,** and **40** (Figure 14).

Complexes **36**–**40** were tested against lung carcinomas A549 and A549/DDP, cisplatin-insensitive cells, and normal healthy (HL-7702) cells. In particular, complex **38** showed the highest cytotoxic activity (IC_50_ = 0.11 μM) against the A549/DDP cell line in comparison with complex **36** (IC_50_ = 3.01 μM), complex **37** (IC_50_ = 0.75 μM), complex **39** (IC50 = 3.87 μM), complex **40** (IC_50_ = 0.91 μM), and cisplatin (IC_50_ = 50.12 μM), all of which showed lower cytotoxic effects against HL-7702 compared with the standard approved regimen based on cisplatin. Complex **37** and, above all, **38,** were the most effective, but the latter led to higher activated Pt content inside the cells (probably because of its greater reduction rate), as well as inside the mitochondria, compared with complex **37** and cisplatin. To explore the mitochondrial effect caused by complexes **37** and **38**, the level of mitochondria membrane potential (MTMP) was monitored. This significantly reduced after **37** and **38** treatments, leading to cell death in lung carcinoma cancer cells due to the induction of mitochondria dysfunction. Thus, the expression levels of the apoptosis-associated proteins were evaluated to clarify their possible mode of action, and it was found that complex **38,** compared with complex **37,** exhibited higher induction in all the proteins, including cytochrome c, active-caspase-3, BAX, apaf-1, and active-caspase-9, thus providing a rational explanation of their relative IC_50_ values. Similar behavior was also observed in in vivo experiments, in which complex **38** was found to be the most cytotoxic of the series. In addition, these new compounds seem to induce minimal side effects in mice, as confirmed by the non-negligible weight loss of the animals after the treatment [53]. Another possible bioactive ligand that could be released by reducing the Pt(IV) complex is an inhibitor of glutathione (GSH) synthesis, which is generally overexpressed in cancer cells or the tumor microenvironment. For example, L-buthionine-(*S*,*R*)-sulfoximine (BSO) is a potent, specific, and irreversible inhibitor of the glutamate–cysteine ligase (GCL), as well as a rate-limiting enzyme in the synthesis of GSH; however, it also possesses antitumor activity in and of itself, although it also has a fast metabolism and excretion issues. Indeed, using this bioactive molecule in axial positions could improve pharmacokinetics and tumor selectivity, especially when used as an albumin-binding moiety, leading to a sensible decrease in GSH levels after its release.

**Figure 15:**
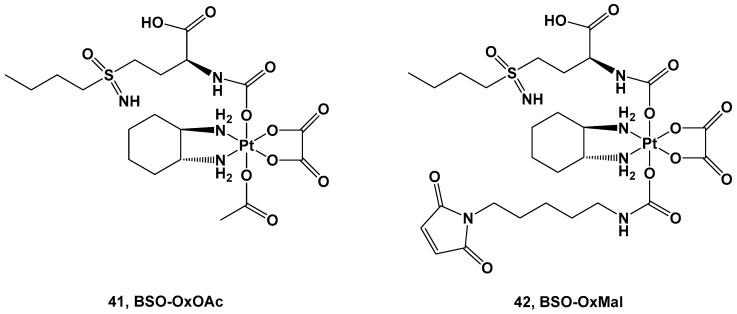
Pt(IV) complexes **41** and **42**.

Two Pt(IV) complexes, derivatives of oxaliplatin, have also been synthesized: BSO-OxOAc (with an inert acetate ligand) and BSO-OxMal (with the albumin-binding maleimide) (Figure 15).

Both complexes were tested against a human colon cancer model (HCT116), an ovarian cancer model (A2780), and their corresponding oxaliplatin (HCT116/OxR) and cisplatin-resistant models (A2780/Cis). Complex **41** was the only compound selected for the in vitro experiments, although its cytotoxicity was reduced in comparison with oxaliplatin and cisplatin in the HCT116 and A2780 cancer cell lines, with IC_50_ = 32.1 μM vs. 0.58 μM and 20.6 μM vs. 1.9 μM, respectively. As such, the resistance mechanisms of the Pt(IV) prodrug (**41**) and its toxicity against nonmalignant cell lines were demonstrated to be extremely negligible, making it a promising candidate for further investigations. For this reason, complex **41** has the advantage of a large therapeutic window but the disadvantage of needing a longer exposure time and more reducing agents to undergo the activation bioprocess.

Conversely, for in vivo experiments, only complex **42** has been explored. Pharmacokinetics studies showed that the AUC and serum half-life of complex **42** increase compared with oxaliplatin, and it is also accompanied by a significantly selective accumulation of the Pt(IV) prodrug in tumor tissue with respect to healthy tissue. Furthermore, both complexes **41** and **42** have been compared with oxaliplatin using in vivo experiments. In the first two weeks, both new complexes and oxaliplatin can reduce the tumor volume, but after this period, only complex **42** proved efficacious in stabilizing the cytotoxic effect, thus prolonging the overall survival of the mice. This higher antitumoral activity could be explained by its stronger GSH-depleting effect, increased DNA damage, and proliferation arrest in comparison with oxaliplatin. In addition, Pt(IV) prodrug did not increase DNA damage in healthy organs, thus confirming that the BSO effect is limited only to tumoral tissue.

**Figure 16:**
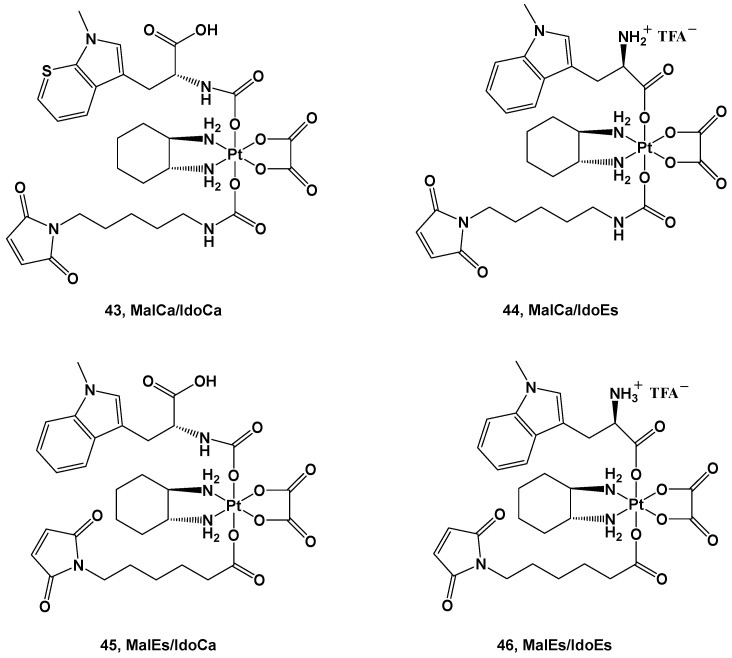
Pt(IV) complexes **43**–**46**.

A structure quite similar to complex **42** was retained in series **43**–**46**; the ligands were an albumin-targeting maleimide and 1-methyl-D-tryptophan (1-MDT) [55]. 1-MDT inhibits the indoleamine 2,3-dioxygenase (IDO) enzyme, which catabolizes tryptophan (Trp) into kynurenine (Kyn), with the binding thereof to its receptor leading to the inhibition of T-cell activation and T-cell proliferation support (Figure 16).

The four derivatives belonging to this series differ from each other in the diverse strategies applied to their synthesis and their reduction behavior.

The advantage of these new complexes seems to rely on the release of an unmodified 1-MDT ligand intracellularly, thus suggesting that coupling this ligand to a platinum(IV) center could be an effective strategy in delivering 1-MDT inside tumoral cells.

**Figure 17:**
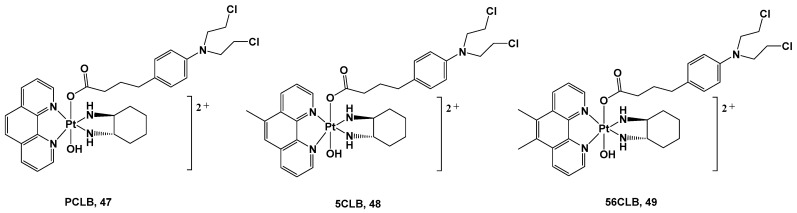
Pt(IV) complexes **47**–**49**.

Furthermore, in vivo experiments have been performed to gain information on their pharmacokinetics and further details on their anticancer activity. The IdoEs complexes, such as **44** and **46**, show higher activity in cell cultures and lower activity in mice because of their faster reduction process, thus leading to deactivation; on the contrary, the IdoCa complexes, such as **43** and **45**, have higher cytotoxic activity in vivo because of their slower activation process, thus retaining their effects until they reach the molecular target. In addition, these experiments have confirmed that these new complexes are capable of inhibiting IDO in malignant tissue, leading to a tumor-specific change in the T-cell population [56]. The choice to employ bioactive axial ligands in the design of platinum(IV) prodrugs could represent a valid strategy in protecting and increasing the efficacy of molecules known to be unstable or easy to deactivate under physiological conditions, thus compromising their use as active ingredients on their own. For example, chlorambucil (CLB) is a potent anticancer agent that can bind the nucleotides of DNA, such as guanine and adenine, at the N(7) and N(3) positions, thus forming DNA crosslinks. Nevertheless, its clinical use is compromised by its low bioavailability and poor selectivity, thus resulting in numerous and serious side effects. For this reason, one possibility is conjugating CLB to a platinum(IV) moiety, resulting in an advantageous delivery system for this drug (Figure 17).

All these complexes were synthesized starting with their Pt(II) precursors, PHENSS, 5MESS, and 56MESS respectively, and were tested against different cell lines and different types of tumors, such as HT29 colon, U87 glioblastoma, MCF-7 breast, A2780 ovarian, H460 lung, A431 skin, Du145 prostate, BE2-C neuroblastoma, SJ-G2 glioblastoma, MIA pancreas, ADDP ovarian (cisplatin-resistant A2780 clone), and nontumor-derived MCF10A breast lines. The cytotoxicity data revealed that complexes **47**, **48**, and **49** exert important antitumor activity, with complex **49** being the most promising in the series. It possessed a potency similar to its Pt(II) precursor, 56MESS, toward all tested cell lines, affording it an average IC_50_ of around 40 μM, but most of all, it proved to be more potent than either cisplatin or CLB. These promising results were confirmed via ^1^H- and ^195^Pt-NMR studies recording the reduction process of Pt(IV) complexes **47**–**49** 5 min after the addition of an appropriate reducing agent with the release of the CLB ligand. This confirms the validity of this synthetic strategy and represents a proof-of-concept for the development of Pt(IV) analogs. However, it is important to mention that these experiments are only approximate and do not necessarily reflect how prodrugs would behave in blood and blood serum.

In addition, complex **49**′s anticancer properties seem to be related to its ability to produce considerable amounts of ROS inside cells, thus promoting an antitumorigenic signal and triggering oxidative stress that can selectively induce cancer cell death. Finally, the presence of the two methyl groups on the phenanthroline ligand could account for the higher cytotoxicity displayed by **49** in comparison with analogs **47** and **48** as a consequence of its higher lipophilicity.

**Figure 18:**
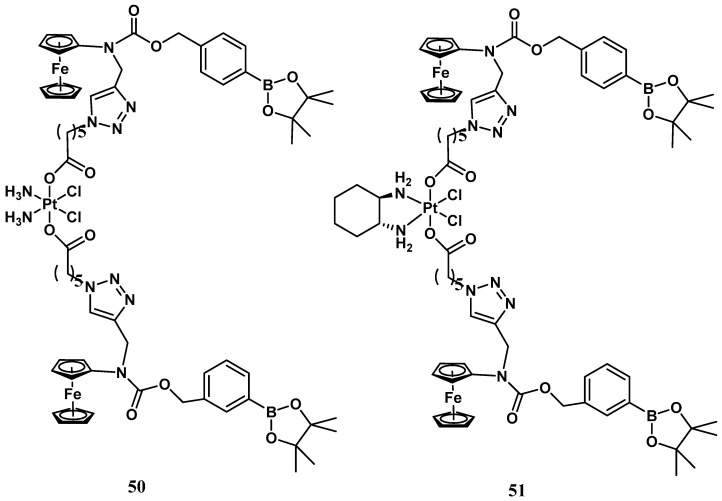
ROS-activated Pt(IV) complexes, **50** and **51**.

Conversely, ROS production is indeed an evident consequence of the increased respiration process, and exaggerated proliferation featuring cancer cells can be usefully exploited as a stimulus for the activation process within tumor tissue. Thus, a ROS-activated platinum (IV) prodrug bearing two boronate-ester-masked N-alkylaminoferrocene ligands has been proposed (Figure 17) [57]. Different studies have revealed that compound **50** can be selectively activated only by ROS, which is resistant to both GSH and ascorbic acid stimuli. The activation pathway presumably provided C-B oxidation, leading to the formation of two N-alkylaminoferrocenes that, once deprotected, may acquire the ability to act as electron donors for the Pt(IV) center, activating the platinum(II) drugs. This prodrug shows comparable antiproliferative activity to cisplatin (IC_50_ = 2.5 μM vs. 2.1 μM) in A2780 cisplatin-resistant human ovarian carcinoma. The corresponding ROS-activated oxaliplatin analog, **51**, bears the same two boronate ester-masked N-alkylaminoferrocenes and was also proposed and evaluated for the same cancer cell line, revealing significant cytotoxicity with an IC_50_ value of 0.4 μM, endowed with a 45-fold smaller effect on healthy cells (Figure 18).

## 4. Conclusions and Further Perspectives

This review summarizes the recent advances in the synthesis of both platinum(II) and prodrugs platinum(IV) complexes for cisplatin-resistant cancer cell lines, whose therapeutic treatment still represents a great challenge. This can be correlated to the lack of selectivity toward the desired target and, therefore, the systemic side effects that often limit their final clinical approval. In this regard, despite continuous efforts in this direction, cisplatin remains the lead compound for so-called orphan tumors. The inability of researchers to bypass these problems can be linked to the incomplete unraveling of the mechanism of action of cisplatin, which is unknown in many ways. In recent years, [58,59,60,61] drug-delivery strategies have been developed to ameliorate the selectivity and, therefore, the systemic effects, but it will be necessary to implement and differentiate molecular targets to reverse therapeutic failure risks. As far as platinum(IV) complexes are concerned, the mechanism of activation and the consequent reduction products that form remain to be clarified. Indeed, the bioactivation in the complex biological system is far from fully addressed, as particular attention needs to be paid to the role played by the protein pool in delineating the activation pathway. Notably, most Pt(IV) prodrugs in the literature have oxygen donor ligands in axial positions, whereas only a few examples comprise different atom donors, which means that, ideally, the vast potential of platinum compounds could be explored. In this sense, a future goal can be achieved by using selective activation and drug delivery systems.

## Data Availability

Data sharing is not applicable. No new data were created or analyzed in this study.
